# Depletion of somatic mutations in splicing-associated sequences in cancer genomes

**DOI:** 10.1186/s13059-017-1337-5

**Published:** 2017-11-07

**Authors:** Laurence D. Hurst, Nizar N. Batada

**Affiliations:** 1 The Milner Centre for Evolution, Department of Biology and Biochemistry, University of Bath, Bath, BA2 7AY UK; 2 Institute for Genetics and Molecular Medicine, University of Edinburgh, Edinburgh, EH4 2XU UK

**Keywords:** Cancer genome, TCGA, ICGC, Mutations, Synonymous, Splicing, Exonic splice enhancers, Negative selection

## Abstract

**Background:**

An important goal of cancer genomics is to identify systematically cancer-causing mutations. A common approach is to identify sites with high ratios of non-synonymous to synonymous mutations; however, if synonymous mutations are under purifying selection, this methodology leads to identification of false-positive mutations. Here, using synonymous somatic mutations (SSMs) identified in over 4000 tumours across 15 different cancer types, we sought to test this assumption by focusing on coding regions required for splicing.

**Results:**

Exon flanks, which are enriched for sequences required for splicing fidelity, have ~ 17% lower SSM density compared to exonic cores, even after excluding canonical splice sites. While it is impossible to eliminate a mutation bias of unknown cause, multiple lines of evidence support a purifying selection model above a mutational bias explanation. The flank/core difference is not explained by skewed nucleotide content, replication timing, nucleosome occupancy or deficiency in mismatch repair. The depletion is not seen in tumour suppressors, consistent with their role in positive tumour selection, but is otherwise observed in cancer-associated and non-cancer genes, both essential and non-essential. Consistent with a role in splicing modulation, exonic splice enhancers have a lower SSM density before and after controlling for nucleotide composition; moreover, flanks at the 5’ end of the exons have significantly lower SSM density than at the 3’ end.

**Conclusions:**

These results suggest that the observable mutational spectrum of cancer genomes is not simply a product of various mutational processes and positive selection, but might also be shaped by negative selection.

**Electronic supplementary material:**

The online version of this article (doi:10.1186/s13059-017-1337-5) contains supplementary material, which is available to authorized users.

## Background

Across tumour genomes, the distribution of somatic synonymous mutations (SSMs) is heterogeneous and commonly thought to reflect differences in transcription, replication timing, chromatin state or DNA repair rate [1–5]. Synonymous mutations that inactivate tumour suppressors [6, 7] can be positively selected. This contrasts with observations within mammalian populations in which some synonymous mutations are typically under negative selection [8–11] and cause disease [12–15]. It is unknown whether such commonplace negative selection on synonymous mutations also occurs within tumours. If it does, then the common assumption that the local synonymous rate is an unbiased estimation of the local mutation rate would require reappraising. This is of substance as identification of driver mutations is commonly done by reference to the local synonymous rate as a means to exclude the possibility of a locally high mutation rate [2].

Recent estimates suggest that 25–45% [16], 30% [17], ~ 60% [18] or 77% [19] of exonic point mutations, synonymous mutations included, lead to splicing disruption. Mutations that affect splicing tend either to be immediately at the splice site or within approximately 70 bp of an exon end [20] where exonic splice enhancers are especially enriched [21] and evolutionarily constrained [8, 9, 22, 23]. As synonymous mutations can, and commonly do, disrupt splicing, we hypothesized that synonymous mutations will be under purifying selection at sites related to splicing in tumours as well. Consistent with this hypothesis, a significant proportion of somatic mutations at exonic ends result in intron retention [24] and cancers are associated with increased rates of alternative splicing [25, 26], although this is in part owing to changes to the profile of ribosomal binding proteins [27]. Here then we sought to test the hypothesis that synonymous mutations affecting splicing are subject to pervasive purifying selection in tumours.

## Results

### Synonymous variants are rare at exon flanks

To detect signals of negative selection, we obtained data from The Cancer Genome Atlas (TCGA) consortium. We selected the top 15 cancer cohorts that had the highest number of samples and the highest average number of synonymous mutations per tumour (see ‘Methods’). We then retained only those point mutations that were synonymous, in part because analysis of synonymous mutations alone mitigates the confounding effect that non-synonymous mutations have on protein function if splicing is unaffected. We refer to these mutations as SSMs.

**Fig. 1:**
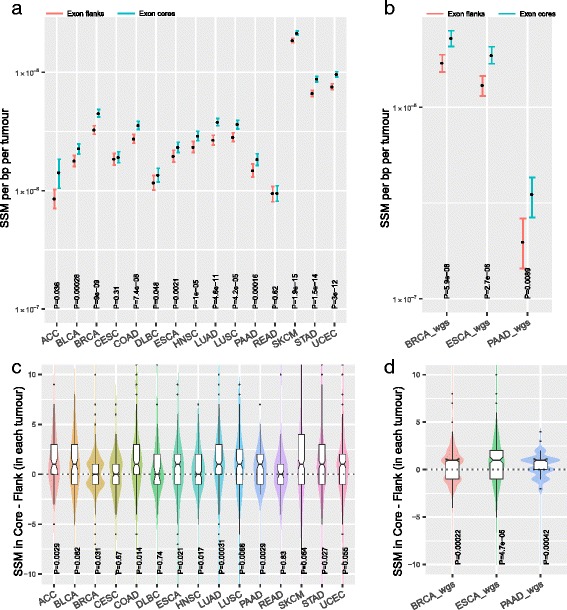
Levels of SSMs at splicing associated sequences are depleted across cancers in both exome and whole-genome sequence (WGS) data. **a**, **b** Levels of SSM at exonic ends and core in the tumours that were exome-sequenced (TCGA) or WGS (ICGC). Y-axis units, synonymous mutations per tumour. Error bars, 95% CI computed by bootstrapping. **c**, **d** Distribution of differences in SSM counts between flank and core within individual tumour that were exome-sequenced (TCGA) or WGS (ICGC). Statistical significance was computed using Wilcoxon signed rank test. The y-axis unit is SSM rate per bp per tumour

We obtained canonical transcripts (i.e. the single transcript with the most supporting evidence) for each known gene for the hg19 human genome assembly from the UCSC Genome Browser. If splicing is important we expect that synonymous variants should be less common at regions near exon ends, ends being where splice-associated mutations are especially enriched [20]. Each of the internal protein-coding exons that are at least 160 bp in length were then partitioned into a 20-bp region from each end (‘flank’) and a 40-bp region from the exon centre (‘core’). Strikingly, the frequency of synonymous variants at the exonic flanks is lower than at exonic cores for tumours in all cancer types analysed (Fig. 1a, Fisher combined *P* = 1.2 × 10^−40^; Cohen’s *d* = 0.19, 95% confidence interval [CI] = [−0.59, 0.97]), with 13 being individually significant and 11 out of 15 being individually significant after Bonferonni correction. One of the two non-significant cohorts (READ, CESC) has only 69 tumour samples, suggesting the lack of significance may be related to small sample sizes.

One possible explanation for the higher density of variants at exon cores is that the exome capture method used by the TCGA consortium might result in higher sequencing depth, and thus more power to detect variants, at exon cores. This we suggest is a priori unlikely to provide an explanation as GC-rich regions are under-covered in exome-sequence data (see e.g. [28]) and exon cores are more GC-rich (Additional file 1: Figure S1). Thus, if anything, any bias might be expected to cause exome-sequencing methods to be conservative as regards the core–flank difference. Nonetheless, to check whether this might affect the results, we obtained data from three International Cancer Genome Consortium (ICGC) studies that performed whole-genome sequencing (WGS) of tumours, WGS having a more uniform coverage [28]. In all instances, the flanks again have significantly lower rates of variants at exon flanks (Fig. 1b; Fisher combined *P* = 1.26 × 10^−11^; Cohen’s *d* = 0.48, 95% CI = [−3.88, 4.84]). The magnitude of the effect appears greater in WGS data. Thus, the observed depletion of SSMs at flanks is unlikely to be an exome capture artefact.

Assuming the exon end depletion reflects splice-associated selection, how common might splice-disrupting mutations be? We here have concentrated on synonymous variants to overcome the evident difficulty in interpretation of results from non-synonymous mutations. Estimates suggest that 25–90% [16–19] of exonic point mutations (synonymous or non-synonymous) lead to splicing defects, although the higher estimates most likely overestimate the frequency of variants that have a selectively relevant effect owing to splicing disruption. If we assume that the variant call rate at exon cores is closer to the ‘true’ mutation rate, we estimate that approximately 17% of all mutational events in exon flanks are unobservable owing to purifying selection. A comparable estimate for the proportion of known disease-associated mutations (from the ClinVar database) that act via disruption of splicing (deduced from the difference between core and flank rates) is striking similar at circa 20%. These estimates assume no exon core mutations disrupt splicing.

An alternative explanation for the observed reduced density of SSM within exonic flanks compared to exonic cores could be differences in alignability or mappability [29] of sequence reads in these two regions. In particular, if sequences from the exonic flanks were less likely to be uniquely mapped compared to reads within the exonic cores, then that could reduce the power to detect mutations. To address this issue, we obtained the ENCODE mappability track for 100 bp which provides a measure of how often the sequence found at the particular location will align back to the genome with up to two mismatches (a perfectly mappable region has a score of 1 while an unmappable region has a score of 0; see ‘Methods’). We find that exonic flanks have a slightly reduced mean mappability compared to exonic flanks (mean mappability for exonic flanks = 0.9795, mean mappability for exonic cores = 0.9810; Mann–Whitney U test *P* value 7 × 10^−7^). We like to note that the relative difference is < 1% and therefore is unlikely to explain the 17% observed reduction in mutation rate between the cores and flanks.

To further rule out potential contribution of technical bias to observed results, we tested for differences in SMM density at the 5’ flank vs the 3’ flank. Prior analysis has suggested that the 5’ end might be the more important one for splice control [30]. If the observed reductions of SSM density in the flank were owing to a systematic bias in exome capture methods specifically reducing coverage and power to call mutations at exon ends, then we would expect that the 5’ and the 3’ flank would have similar reduction in SSM density. However, consistent with the expectation that the negative selection is acute at splicing regulating sequences and further ruling out technical artefact, we observe that SSM density at 5’ flanks tends to be lower than in 3’ flanks in both exome and WGS data of tumours with in exome data 14 of 15 tumours having a lower SSM density at 5’ flanks than at 3’ flanks (binomial test *P* < 0.001; Additional file 1: Figure S2).

The above analyses pooled all data from a given tumour type. However, there is extensive heterogeneity in mutation rates between manifestations of the same tumour class. To determine if the previously observed effect is in individual tumours as well, we computed the difference in SSM density at flank vs core in a paired manner for each individual tumour. As expected, tumours with more variants called in exon cores tend to be tumours with more variants called at exon flanks (Additional file 1: Figure S3). Importantly, examining the distribution of paired core–flank differences, we again observe a systematic trend for exon flanks to have fewer variants than exon cores in both exome (Fig. 1c, Fisher test *P* = 1.87 × 10^−10^) and WGS analyses (Fig. 1d, Fisher test *P* = 1.66 × 10^−9^).

Mutations at canonical splice sites (normally defined as the 2 bp in intron and 2 bp of the exon boundary representing splice donor and acceptor sites) are well-known to be deleterious. To test if the observed reduction is simply a consequence of reduced SSM at canonical splice sites, we repeated all the analyses by only considering regions not containing these splice sites (i.e. the three nucleotides of each exonic end); we see similar results for both TCGA exome data and ICGC WGS data (Additional file 1: Figure S4).

### No evidence that differential mutability explains depletion of SSM in flanks

**Fig. 2:**
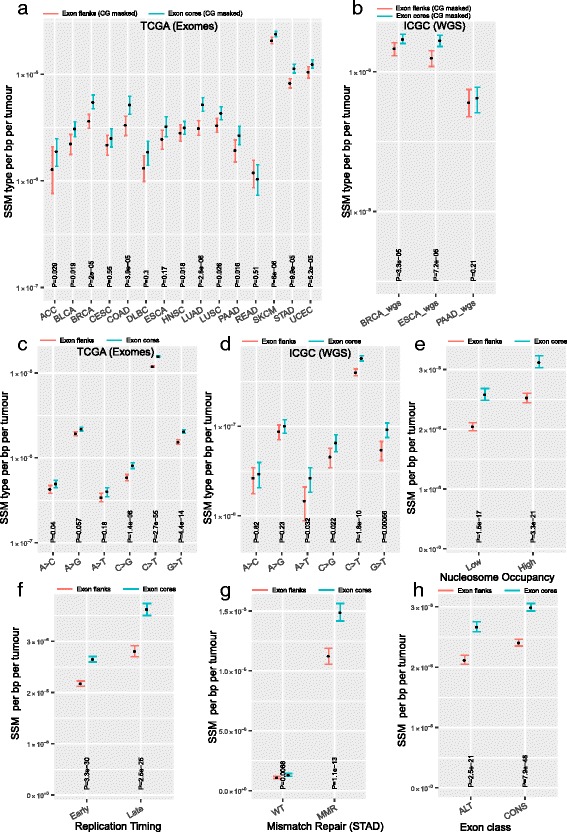
Synonymous rates are lower at exon flanks compared with cores when controlling for multiple variables. **a**, **b** Comparison of SSM mutational load between exon flanks and cores in which CpG sites were masked out. **c**, **d** SSM from TCGA exomes (**c**) and ICGC WGS (**d**) were segregated into six equivalent classes of substitutions and normalized by the number of reference nucleotides. **e** SSM relative to nucleosome occupancy. Top 25% (bottom 25%) of the nucleosome occupied regions are designated as high (low). **f** SSM relative to DNA replication timing. **g** SSM relative to mismatch repair status in TCGA STAD. **h** SSM comparison in alternative vs constitutive exons. Statistical significance was computed using Wilcoxon signed rank test. The y-axis unit is SSM rate per bp per tumour. Error bars, 95% CI computed by bootstrapping

The above results are consistent both with a model, which we refer to as the Selection Model, that suggests increased purifying selection at exon ends (and a uniform mutation rate across exons) and also with a model, which we refer to as the Mutation Model, which suggests increased mutation rates at exon cores (and uniform or absent selection). The latter is a viable model in that GC content tends to be higher in exon core, while AT content is higher at exon flanks (Additional file 1: Figure S1) (N.B. exonic splice enhancer motifs enriched at exonic ends are greatly enriched for purines, adenine in particular). Given the hypermutability of cytosines in the CG context, the Mutation Model is a reasonable null model. We therefore masked out all the CG dinucleotides in the flanks and cores and recomputed the SSM density (Fig. 2a, b). The effect remains significant (Fisher’s method, *P* = 4.62 × 10^−8^; for Fig. 2a, Cohen’s *d* = 0.23, 95% CI = [−0.54, 1.01] and for Fig. 2b, Cohen’s *d* = 0.23, 95% CI = [−0.38, 4.97]) indicating that the core–flank difference cannot be accounted for in totality in terms of different CG contents causing different mutation rates. To determine if only a certain class of substitutions were depleted, we partitioned all SSMs based on the six canonical substitution types and normalized call rates per appropriate nucleotide content (for example, we divided SSM^A>C^ by number of A and number of T nucleotides as A > C is equivalent to T > G). After normalization, we still observe that core has a higher variant rate than flank in both exome data with four out of six substitutional types being individually significantly depleted in flanks (Fig. 2c, combined *P* value, Fisher’s method, *P* = 1.49 × 10^−15^; Cohen’s *d* = 0.15, 95% CI = [−1.31, 1.61]) and WGS data (Fig. 2d, Fisher’s method, *P* = 1.86 × 10^−8^; Cohen’s *d* = 0.24, 95% CI = [−1.22, 1.71]). We note that it is expected that the patterns of somatic substitutions differ significantly between the TCGA exome data (Fig. 2c) and ICGC WGS (Fig. 2d) because the majority (71%) of the tumours in the latter set represent breast cancers [31]. Breast cancers not only have relatively low mutation rates but different tumours from patients of the same cancer type can have distinct patterns of mutation reflecting underlying biological mechanisms contributing to DNA damage [32]. To further test if mutational biases can explain observed differences in levels of SSMs between exon flanks vs cores, we quantified the mutations at 96 canonical trinucleotides (trinucs) formed by including a base before and after each SSM. As we are quantifying mutations only at a small fraction of exonic regions for the core and the flank regions, partitioning of SSMs into 96 trinucs is expected to result in very few trinucs in a majority of tumours. Consequently, the confidence intervals are large and the majority of the differences are non-significant (Additional file 1: Figure S5). For the differences that are significant, the mutation burden at flanks is lower than in the cores in six of seven incidences. Although the nature of this analysis is underpowered to detect true differences, the results in Additional file 1: Figure S5 are not inconsistent with the results shown in Fig. 2c and d.

Nucleosomes are also thought both to potentially be enriched near exon ends [33, 34] (possibly to determine splicing) and to modulate mutation rates [35]. To account for potential differences in nucleosome occupancy (which correlates with DNA accessibility) between flank and core, we separated exons within genomic regions that have high nucleosome occupancy, which are regions with closed or heterochromatin, from those in low nucleosome occupancy, which are regions with open or accessible chromatin. The difference between core and flank is not explained by differences in nucleosome occupancy (Fig. 2e). Nor is the difference explained by replication timing [2] (Fig. 2f), or mismatch repair deficiency [1, 4] (Fig. 2g), supporting the view that the depletion is not a consequence of mutational heterogeneity. We observe that the difference is seen in both constitutive and alternative exons (Fig. 2h). Just as alternatively spliced exons can have especially low synonymous substitution rates [36], an effect that seeps into the flanking intronic sequence [36], so too are synonymous variants rarer in alternative exons. These results remain after masking out CG dinucleotides (Additional file 1: Figure S6). A parsimonious interpretation of these data is thus more common purifying selection in alternative exons in both populations and tumours.

### Depletion of synonymous variants is acute in exonic splicing enhancer motifs

To obtain further evidence that the SSM depletion is associated with splicing, we analysed SSM levels in exonic splice enhancers (ESE), which are hexamers that are strongly implicated in splicing [37]. We used a list of 84 ESEs made from intersection of multiple independent ESE datasets (the INT3 dataset), thus expected to have a low false-positive rate but potentially a high false-negative rate. To mitigate the false-negative problem, we defined non-ESEs as the subset of 4096 hexamers that are > 2 edit-distance away from ESEs. We identified regions in canonical exons where the two sets aligned perfectly. We identified ~ 72 K ESE regions and ~ 230 K non-ESE regions in the coding exons of the human genome. As expected, nucleotide content is different between ESE and non-ESE (Additional file 1: Figure S7).

**Fig. 3:**
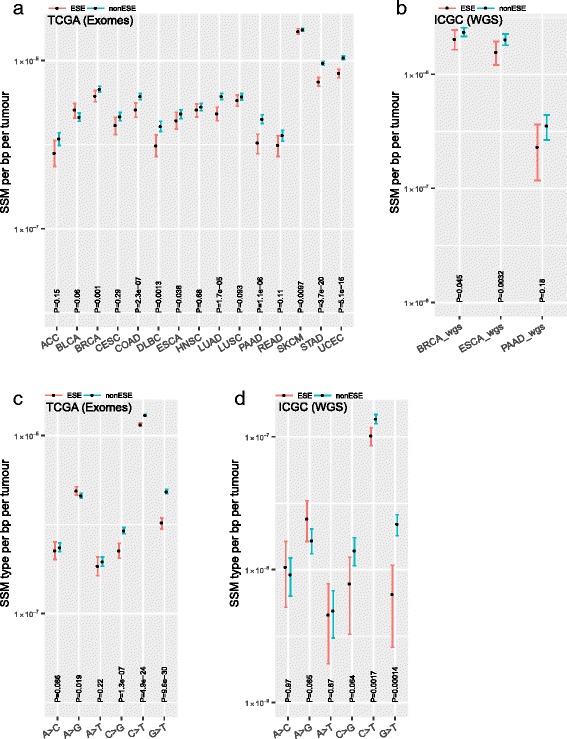
Levels of somatic synonymous mutations in ESEs. **a**, **b** Levels of SSM at ESE and non-ESE (see ‘Methods’) in the tumours that were exome-sequenced (TCGA) or WGS (ICGC). Y-axis units, synonymous mutations per tumour per. **b**, **c** SSM from TCGA exomes (**a**) and ICGC WGS (**b**) were segregated into six equivalent classes of substitutions and normalised by the number of reference nucleotides. Statistical significance was computed using Wilcoxon signed rank test. The y-axis unit is SSM rate per bp per tumour. Error bars, 95% CI computed by bootstrapping

For both classes we computed the density of synonymous variants. We find that the density within the ESEs is lower than the rate observed in non-ESE in all but one of 15 cancers (Fig. 3a; Cohen’s *d* = 0.20, 95% CI = [−0.58, 0.97]), the effect being significant in nine (Fisher’s method, *P* < 1.86 × 10^−25^). A similar effect is observed in somatic mutations identified from WGS (Fig. 3b; Fisher’s method, *P* = 0.0018; Cohen’s *d* = 0.29, 95% CI = [−4.03, 4.62]), thus discounting possible technical artefacts of subset capture involved in exome sequencing that may contribute to the observation. Given the different nucleotide contents in ESE and non-ESE (Additional file 1: Figure S7), we also scrutinized individual classes of nucleotide. This too supports the lower rate of observed mutation in true ESE in exome data (Fig. 3c; Fisher’s method, *P* = 9.7 × 10^−14^; Cohen’s *d* = 0.14, 95% CI = [−1.31, 1.61]) and WGS data (Fig. 3d; Fisher’s method, *P* = 0.0003; Cohen’s *d* = 0.17, 95% CI = [−1.28, 1.64]). We conclude that splicing-associated sequences have reduced SSM density.

To further test if mutational biases can explain observed differences in levels of SSMs between ESE and non-ESE, we quantified the mutations at 96 canonical trinucs as before. Given that we are assessing mutations at a small fraction of exonic region, partitioning of SSMs into 96 trinucs is expected to result in very few types of trinucs in a majority of tumours. Consequently, the confidence intervals are large and majority of the differences are non-significant (Additional file 1: Figure S8). There were 39 significant differences out of which there were 29 trinuc in which the mutation rate at ESE is lower than at non-ESE. Overall, the results (Additional file 1: Figure S5 and Additional file 1: Figure S8), though underpowered, are consistent with our conclusion that mutational biases are insufficient to explain the observed differences in SSM in outer/flank or ESE/non-ESE regions.

### Reduced SSM is not restricted to essential or cancer-associated genes

**Fig. 4:**
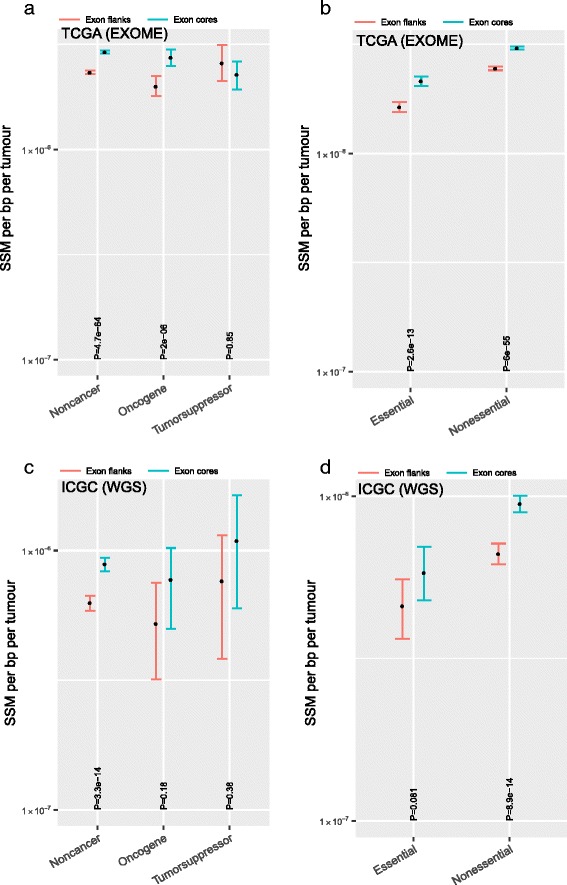
Reduced levels of SSM at exon flanks are seen in all gene classes except in tumour suppressors. **a**, **c** SSM levels at flank and core regions of exons from oncogenes, tumour suppressors and non-cancers using TCGA exomes (**a**) or ICGC WGS (**b**) data. **b**, **d** SSM levels at flank and core regions of exons from essentials and non-essentials genes defined by CRISPR/Cas9 screen using TCGA exomes (**b**) or ICGC WGS (**d**). Statistical significance was computed using Wilcoxon signed rank test. The y-axis unit is SSM rate per bp per tumour. Error bars, 95% CI computed by bootstrapping

One possible reason why synonymous somatic mutations affecting splicing might be deleterious is because they compromise the function of genes that are required for tumour proliferation or survival. Accordingly, a prediction is that oncogenes would show depletion of SSMs while tumour suppressors would show either enrichment due to positive selection or no depletion. We partitioned genes into oncogenes and tumour-suppressors based on annotation from the Cancer Gene Census database [38]. Consistent with expectation, SSMs are significantly depleted in oncogenes but not in tumour suppressors (Fig. 4a); interestingly, however, non-cancer genes also show depletion.

A possible reason for depletion of SSMs in non-cancer genes is that the set of non-cancer genes contains unidentified cancer genes and/or essential genes, such as housekeeping genes (i.e. cell cycle regulation, DNA replication, transcription, translation or metabolic and others) that are required for proliferation of any cell, not just cancer cells. This would predict that the depletion of variants at exon ends should be observed in non-cancer essential genes but not in non-cancer non-essential genes. We partitioned non-cancer genes into essential and non-essential defined by genome-wide functional genomics screen in a human cell line [39]. We found depletion of SSMs in both essential and non-essential genes, to an approximately equal magnitude (Fig. 4b). The same trends are seen in WGS data (Fig. 4c, d), except that the oncogene difference is no longer significant. In neither data class are tumour suppressor genes significantly different in core and flank and the two datasets are inconsistent as to whether core rate is higher than flank rate. We conclude that the depletion of SSMs in tumour genomes is unlikely to be explained solely by positive selection of mutations (i.e. in tumour suppressors) that promote tumour proliferation.

## Discussion

While it is classically presumed that tumour development is dominated by positive selection with negligible purifying selection, here we have presented evidence that synonymous mutations in tumours might be under purifying selection. Consistent with the deleterious effect of SSM on pre-messenger RNA splicing, the affect is especially acute, not just at the 20-bp region near the exon boundary, but also at ESEs. Jung et al. [24] have analysed RNA-sequencing data from TCGA across cancer types and have found that substitutions (both synonymous and non-synonymous) within exonic flanks up to 30 bp from the exon boundaries can cause aberrant splicing; this work is consistent with our conclusion that there is negative selection in this region.

### Mutation bias or selection?

The pattern that we describe, a lower rate of SSMs at exon flanks compared with exon cores, could in principle be accounted for in terms of a core-flank mutation bias alone (i.e. without having to evoke purifying selection). Several lines of evidence argue against this, but are not definitive. First, in somatic cells, methylation is most common at exon flanks [40]. As a consequence, the absolute mutation rate (rather than the observed SSM density) should be higher at exon flanks. Indeed, a similar disparity explains why exons evolve faster than introns at synonymous sites [41]. All things being equal, we thus expect a higher not a lower SSM rate at flanks. This makes our test conservative. Second, the core–flank difference is seen in all cancers. As it is known that different cancer types have enrichment of different types of mutations [32], it is not obvious why the observed depletion should be seen in all cancers (see panels a and b of Figs. 1, 2 and 3). Third, we have shown that the effect is robust to control for known correlates to the mutation rate including nucleosome occupancy, replication timing and DNA repair, all of which provide coherent null models that could explain mutation rate variation. However, intragene variation in nucleosome occupancy is not controlled for. Fourth, we have shown that the effect is especially acute in ESEs. Indeed, when we consider all possible nucleotide triplets, we observe significantly more in ESEs with a lower rate in the flanks than the core, suggesting that we are not observing a simple nucleotide-dependent mutation bias. Fifth, as expected under a selection model, the lower rate is not seen in tumour suppressors, as this is the one class of gene within which purifying selection in tumours is not expected. However, this could simply imply that this class are the uniquely different set of genes as in these there might be positive selection for splice disruption. Sixth, while 5’ and 3’ exon ends have similar nucleotide usage [42], they differ in the extent to which they control splicing [30] and differ also in the flank–core reduction. Seventh, just as purifying selection on synonymous mutations is most acute in alternative exons, so too is SSM density lower in alternative exons (even allowing for differential CpG density).

Given the above, just as a low Synonymous Nucleotide Polymorphism (SNP) rate at exon flanks in circulating SNPs [23, 43], but not at disease-causing SNPs, is consistent with purifying selection at synonymous sites, so too are our data consistent with the same model. Assuming the exon end depletion reflects splice-associated selection, how common might splice-disrupting mutations be? We here have concentrated on synonymous variants to overcome the evident difficulty in interpretation of results from non-synonymous mutations. Estimates suggest that 25–90% [16–19] of exonic point mutations (synonymous or non-synonymous) lead to splicing defects, although the higher estimates most likely overestimate the frequency of variants that have a selectively relevant effect owing to splicing disruption. If we assume that the variant call rate at exon cores is closer to the ‘true’ mutation rate, we estimate that approximately 17% of all mutational events in exon flanks are unobservable owing to purifying selection. A comparable estimate for the proportion of known disease-associated mutations (from the ClinVar database) that act via disruption of splicing (deduced from the difference between core and flank rates) is striking similar at circa 20%. These estimates assume no exon core mutations disrupt splicing. Conservative direct estimation of the proportion of disease-associated mutations that act via splicing [44] suggests a lower figure of ~ 10%. Either way, the estimate of ~ 17% is within the same bounds and so should not be considered in any manner unexpectedly high.

Despite the parallels with population-level results and implication of synonymous mutations in disease, it is near impossible to prove the absence of a mutation bias of unknown origin. One might indeed note that the flank–core difference that we observe in tumours is not the same for all nucleotides (Fig. 3c and d). The reasons for this are not clear. Indeed, ESEs are purine rich (A and G) and these two nucleotides appear to be differently affected. Further, we see no evidence that the depletion is more acute in more strongly expressed genes and no evidence that intron flanks and cores have different SSM depletion, both of which argue for the exon end effect to reflect a hidden mutation bias (data not shown) (note that evolutionary analysis suggests the terminal 20 bp of introns evolve more slowly than intron cores [45]).

It is, moreover, valid to ask why purifying selection is not routinely observed in tumour genomes. This conclusion in part results from the fact that Ka/Ks < < 1 is rarely seen. This, however, is problematic as Ka/Ks is not well suited to analysis of lineages with recent common ancestors as time for purging of weakly deleterious non-synonymous mutations is not long enough [46]. Similarly, testing for purifying selection by examination of reduced frequency of nonsense mutations is problematic as this fails to allow for the fact that heterozygous nonsense mutations can be buffered by nonsense mediated decay. Nonetheless, the patterns that we observe, while consistent with purifying selection of a mode similar to that seen over evolutionary time, is not definitively shown to be owing to purifying selection. If there are mutational biases that we have failed to consider (possibly associated with epigenetic marks peculiar to ESEs and exon ends) that are in turn differentially mutagenic, then such effects could, in principle, explain our data. Thus, we claim to have identified an unusual depletion of SSMs at exon ends and in ESEs that could be explained by purifying selection owing to splicing disruption. We do not claim that we have demonstrated this beyond all reasonable doubt.

### If it is selection, how might this operate?

Assuming that some of the observed depletion of SSMs is indeed owing to splicing-related purifying selection, we would like to suggest the following reasons as plausible causes for this selection. The obvious first reason is loss-of-function of a gene due to truncation or absence of functional protein domains which are required for cell survival, proliferation, metabolism and various housekeeping functions. The observation of the core–flank difference in non-essential non-cancer genes suggests this is not the full explanation. In addition, that nearly all gene classes show the same depletion argues for a hidden mutation bias. A second reason is gain-of-function. The splicing defect could lead to expression of an alternative transcript isoform whose product is toxic or cell-fatal (possibly because it leads to or restores apoptosis). Cells expressing such mutations fail to contribute to tumour mass. A third reason could be immune-editing [47]. The altered splice isoform of the gene could express peptides at the 3’ end that can serve as neoorfs. Such neoorfs can be presented on MHC 1 and lead to immune-mediated elimination of the cell harbouring that mutation. This later model could explain why just about every class of gene is affected. Functional studies are required to unambiguously estimate the relative contributions of these causes. In certain cases, splicing mutations can persist: if the gene is not expressed; or the resulting isoform does not have a negative functional consequence or is efficiently degraded by the nonsense mediated decay pathway or is in tumour suppressor genes whose inactivation supports tumour growth and is thus positively selected [6].

Notice that in the above we have presumed that mutations that we do and do not see are largely owing to effects within the tumour itself. This is not to claim that mutational processes are necessarily different in tumours and normal somatic cells. Indeed, no mutational process that is cancer-specific has been described and there is no obvious reason as to why DNA damage due to exogenous stress (such as ultraviolet irradiation or cigarette smoke) would not be similar in normal somatic cells and transformed cells. However, cancer cells, due to their high proliferation, do have elevated levels of DNA damage which are thought to arise from metabolic by-products and replication stress. Moreover, somatic cells with elevated DNA damage (which would be more likely to have intact DNA damage response pre- rather than post-transformation) undergo cell cycle arrest or cell death in case of excessive damage. By contrast, due to high mutational load and mutations in tumour suppressors such as P53, tumours tend to be defective in apoptosis and so endure and accumulate (‘passenger’) mutations. While it is not clear that this might affect the intragene distribution of SSMs, it follows that post-transformation mutations probably make up a majority of the somatic mutations in the cancer genome data. This argument suggests that the TCGA cancer somatic mutation list is likely to be dominated by mutations post transformation and as a result the signals observed here are likely coming from tumour cells.

### Implications for the study of cancer

What are the implications of our study? These results suggest that either the sampled mutational spectrum of cancer genomes may not simply be a product of various mutational processes and positive selection, but is also shaped by negative selection, or that the profile of mutations has finely grained variation that is currently poorly understood. Either way, attempts to infer positive selection in tumours under the assumption that the local density of synonymous variants provides an unbiased estimator of background rates could lead to misinference. The method is comparable to the Ka/Ks ratio employed in cross-species analysis to search for genes and domains under positive selection. This method runs under the supposition that when the rate of protein evolution (Ka) exceeds the rates of background evolution (for which Ks, the synonymous rate, is a proxy), that this is most likely owing to positive selection. If purifying selection affects synonymous mutations then the local mutation rate will be underestimated. If the reduced SSM rates are owing to highly regionalised mutation rate reductions, then the definition of the ‘local’ mutation rate is contingent on what one means by ‘local’.

Between species analysis suggests that the former issue may be profound as searches for domains with Ka/Ks > 1 identify many more locations where there is strong purifying selection on synonymous sites than it finds sites under positive selection at the protein level [48]. These Ka/Ks > 1 domains with locally low Ks tend to be alternative exons [48], regions that we also observed to have an unusually low SSM density. If the depletion that we have observed is owing to purifying selection then one improvement would be to restrict analysis of the background rate to synonymous sites at exon cores in sequence that does not specify ESE and RNA-binding protein motifs, as these too are under purifying selection [49]. However, if the depletion is owing to a hidden mutation bias, then we need to understand it to enable appropriate control.

## Conclusions

Exon ends and splicing-associated motifs have a low frequency of synonymous mutations in cancers. This is consistent with either (1) purifying selection against splice disrupting mutations or (2) a mutation bias of unknown cause that causes lower mutation rates at exon ends, in splicing-associated motifs and in a manner that differentially affects constitutive and alternative exons. Either way, these findings have implications for modelling somatic mutations during cancer evolution, identifying additional splicing-associated sequences, functional annotation of synonymous somatic variants and identification of cancer-driving mutations.

## Methods

### Data source

TCGA tier 3 filtered somatic mutations (relative to hg19 human genome assembly) called from exome sequence data were downloaded from the Broad GDAC Firehose (date stamp 20160715). As the estimate of synonymous mutation rate is done over a small interval (20 bp), we chose to select a subset of TCGA cohorts that had sufficiently high mutational load and also had sufficient number of samples. The list of cohorts used were the union of the following two set of cohorts: (1) top dozen cohorts with the highest average synonymous mutation load per tumour; and (2) top dozen cohorts with the highest number of synonymous mutations data points (i.e. number of tumours in the cohort times the average number of synonymous mutations per tumour). The acronyms for the various cancers in TCGA are as follows (n = number of samples, m = average total mutation load per tumour within the exomic region captured): ACC (n = 90, m = 223) = adrenocortical carcinoma; BLCA (n = 130, m = 302) = bladder urothelial carcinoma; BRCA (n = 977, m = 92) = breast invasive carcinoma; CESC (n = 194, m = 239) = cervical and endocervical cancers; COAD (n = 460, m = 154) = colorectal adenocarinoma; DLBC (n = 48, m = 352) = diffuse large B-cell lymphoma; ESCA (n = 185, m = 315) = oesophageal carcinoma; HNSC (n = 279, m = 185) = head and neck squamous cell carcinoma; LIHC (n = 198, m = 140) = liver hepatocellular carcinoma; LUAD (n = 230, m = 315) = lung adenocarcinoma; LUSC (n = 178, m = 361) = lung squamous cell carcinoma; PAAD (n = 150, m = 202) = pancreatic adenocarcinoma; READ (n = 319, m = 69) = rectum adenocarcinoma; SKCM (n = 343, m = 846) = skin cutaneous melanoma; STAD (n = 289, m = 513) = stomach adenocarcinoma; UCEC (n = 248, m = 744) = uterine corpus endometrial carcinoma. We downloaded annotated cancer mutations (‘simple somatic mutations open’) identified from WGS data from the ICGC DCC (dcc.icgc.org). We used data from three published studies: (1) BRCA_wgs, represents data from 560 breast cancers [31]; (2) ESOP_wgs, represents data from 129 cases of oesophageal adenocarcinoma [50]; and (3) PANC_wgs, represents data from 100 cases of pancreatic ductal adenocarcinoma [51]. For both TCGA and ICGC substitution mutation, we defined synonymous and non-synonymous mutations based on change in amino acid sequence. All analysis was done using SSMs only. The list of STAD samples associated with Microsatellite instability (MSI), and therefore mismatch repair-deficient, were obtained from supplementary materials associated with the TCGA STAD publication [52].

Bam formatted file indicating nucleosome occupancy data for GM12878 cells was obtained from UCSC Encode portal (http://hgdownload.cse.ucsc.edu/goldenPath/hg19/encodeDCC/wgEncodeSydhNsome/). List of canonical genes was obtained from UCSC genome browser (http://hgdownload.soe.ucsc.edu/goldenPath/hg19/database/knownCanonical.txt.gz). DNA replication timing for IMR90 cells was taken from Hansen et al. [53]. List of exonic splice enhancers, INT3, were obtained from Caceres and Hurst. EST-based classification of exons into alternative and constitutive spliced categories was taken from the HexEvent database [54]. Classification of genes into oncogenes and tumour suppressors was based on the data obtained from the Cancer Gene Census (http://cancer.sanger.ac.uk/census/) [38]. Classification of genes into essential and non-essential was based on genome-wide CRISPR/Cas9 screen in KBM7 cells [39]. The 100-bp mappability track [29] from hg19 human genome assembly was obtained from the UCSC genome browser (ftp://hgdownload.soe.ucsc.edu/goldenPath/hg19/encodeDCC/wgEncodeMapability/wgEncodeCrgMapabilityAlign100mer.bigWig).

### Data processing

All manipulation of mutations and genomic intervals were done using custom Python scripts that employed Samtools and BEDTools. We generated intervals near the ends and centre of exons from all the human canonical exons obtained from the UCSC genome browsers. Only internal (i.e. not 5’ UTR or 3’ UTR) exons > 160 bp were considered. For each exon, we generated a list of intervals (*L*, 20) or (4, 20), (*L*-20, l), (*L*-24, *L*-4), where all the positions are relative to the exon start and *L* is the length of the exon, were designated to be ‘flank’. Similarly, regions (*M*-20, *M*) and (*M*, *M* + 20), where *M* is the centre position of the exon, were designated as exon ‘core’. The flank and the core interval set each spanned 1,727,320 bp total.

For each exon (≥160 bp), we identified the location of ESEs defined by the INT3 database. Only exons from genes on the Watson (+ strand) were retained due to computational considerations. We generated non-ESEs by generating all possible hexamers (n = 4^6^) and discarding those that had one or fewer nucleotide difference from ESE. Exonic regions in the range of 1–69 bp, which are known to harbour splice-associated sequences, were used to identify location of ESE and non-ESE hexamers. Exonic regions matching ESE, that overlapped with exonic regions matching non-ESE, were discarded.

Single nucleotide substitutions were separated into the following six possible classes: (1) A > C; (2) A > G; (3) A > T; (4) C > G; (5) C > T; (6) G > T. Custom scripts were written to find the one nucleotide before and after context of each SSM.

Exons with nucleosome occupancy in the top 25th percentile were designated as high-nucleosome occupied and those with nucleosome occupancy in the bottom 25th percentile were designated as low-nucleosome occupied. Nucleosome occupancy data (wgEncodeSydhNsomeGm12878AlnRep1) were obtained from ENCODE.

### Statistical analysis

All statistical analysis and figure preparation was done using *R*. We used the function smean.cl.boot from the package Hmisc in R to compute the bootstrap CI, with *B* = 500. Cohen’s d and its CI was computed using the compute.es package in *R*.

## Additional file

Additional file 1: Supplementary **Figures S1–S8.** (PDF 1831 kb)

## Abbreviations

ICGC: International Cancer Genome Consortium
SSM: Somatic synonymous mutation
TCGA: The Cancer Genome Atlas
WGS: Whole-genome sequence
