# Supplementary material for: Depletion of somatic mutations in splicing-associated sequences in cancer genomes
Source: Genome Biol. 2017 Nov 7;18:213. doi: 10.1186/s13059-017-1337-5 (PMC5678748; doi:10.1186/s13059-017-1337-5)

Supplementary Figures

Figure S1. **Overall nucleotide content of flank and core exonic regions**.

Figure S2. SSM density in 5' versus 3' exon flanks. A) TCGA exome data. B) ICGC whole genome sequencing data.

Figure S3. Scatter plot of SSM density in flank versus core regions in individual tumours. For each tumour, levels of SSM in flank and core regions were computed. Green line represents y=x line and blue line represents least square fit ("lm" in R) with grey band representing 1 SEM.

Figure S4. **Comparison of SSM density in flank region adjacent to canonical splice sites compared to exonic cores.** We repeated the analysis shown in Figure 1a and 1b but did not include canonical splice sites (i.e. the 3 nucleotides from the ends of each exon) for both TCGA exomic data (**a**) and ICGC WGS data (**b**). Statistical significance was computed using Wilcoxon Signed Rank test. The y-axis unit is SSM rate per bp per tumour. Error bars, 95% confidence interval computed by bootstrapping.

Figure S5. Comparison of SSM density between flanks and cores for 96 trisnps. For this analysis we considered tumour samples from top 12 highly mutated cancer types (SKCM, UCEC, STAD, COAD, LUSC, BLCA, LUAD, READ, ESCA, PAAD, CESC, HNSC). For each SSM in the flank or the core region, we identified it's a nucleotide before and after (i.e. trisnp). For each tumour that had atleast 5 trisnps, we quantified the frequency of each of the 96 possible trisnps. The y-axis unit is frequency of each trisnp per tumour. Error bars, 95% confidence interval computed by bootstrapping.

Figure S6. SSM density in Alternate versus Constitutive exon flanks in which CG dinucleotides are masked out. Similar to Figure S2 except that flanks were partitioned into Alternative or Constitutive using classification given in HexEvent database.

Figure S7. **Overall nucleotide content of ESE and non-ESE exonic regions**. ESE motifs from the INT3 database were identified in each exon as described in methods.

Figure S8. Comparison of SSM density between ESE and non-ESE for 96 trisnps. Similar to Figure S5, for this analysis we considered tumour samples from top 12 highly mutated cancer types (SKCM, UCEC, STAD, COAD, LUSC, BLCA, LUAD, READ, ESCA, PAAD, CESC, HNSC). For each SSM in the ESE or the non-ESE region, we identified it's a nucleotide before and after (i.e. trisnp). For each tumour that had atleast 5 trisnps, we quantified the frequency of each of the 96 possible trisnps. The y-axis unit is frequency of each trisnp per tumour. Error bars, 95% confidence interval computed by bootstrapping.

Figure S1

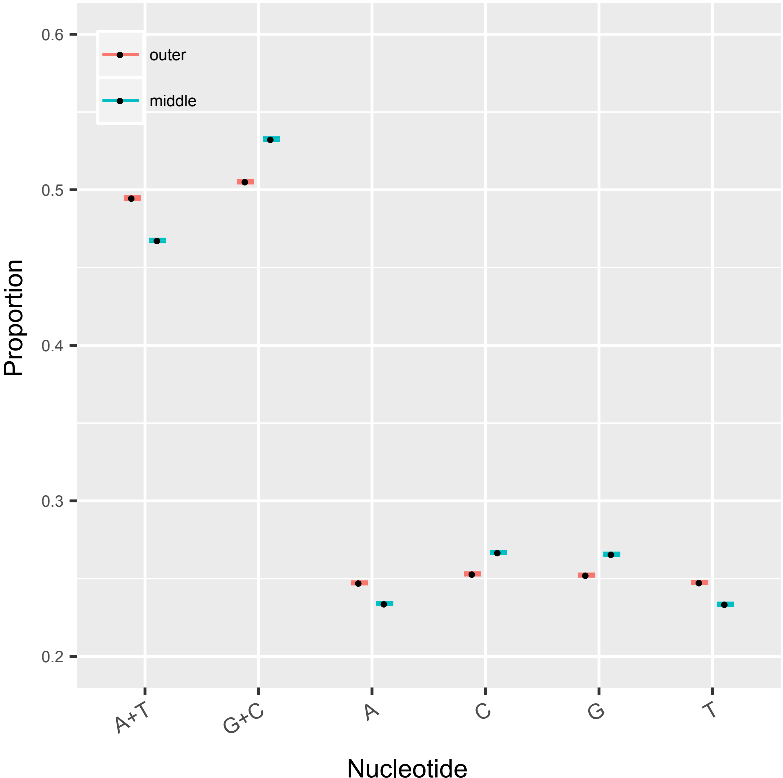

Figure S2

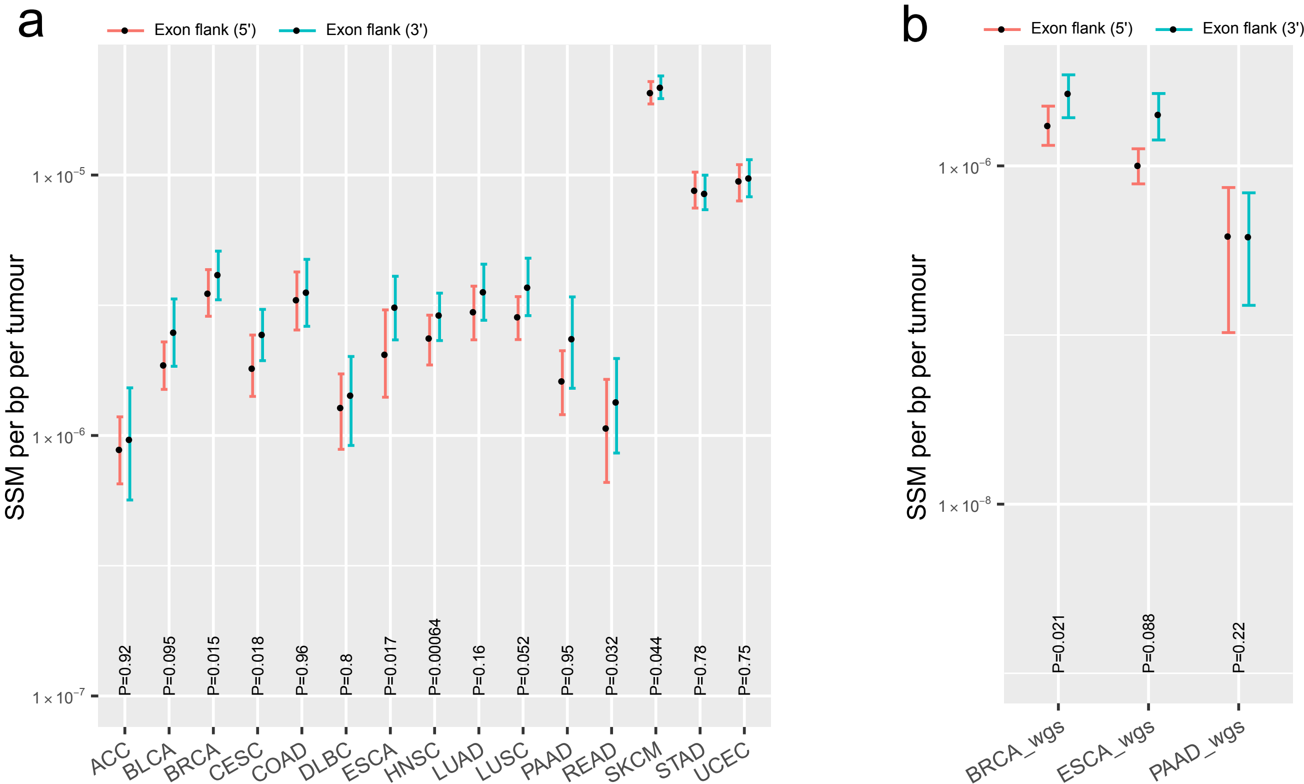
a
Exon flank (5')
Exon flank (3')
SSM per bp per tumour
1×10^{-5}
1×10^{-6}
1×10^{-7}
P=0.92
P=0.095
P=0.015
P=0.018
P=0.96
P=0.8
P=0.017
P=0.00064
P=0.16
P=0.052
P=0.95
P=0.032
P=0.044
P=0.78
P=0.75
ACC
BLCA
BRCA
CESC
COAD
DLBC
ESCA
HNSC
LUAD
LUSC
PAAD
READ
SKCM
STAD
UCEC
b
Exon flank (5')
Exon flank (3')
SSM per bp per tumour
1×10^{-6}
1×10^{-8}
P=0.021
P=0.088
P=0.22
BRCA_wgs
ESCA_wgs
PAAD_wgs

Figure S3

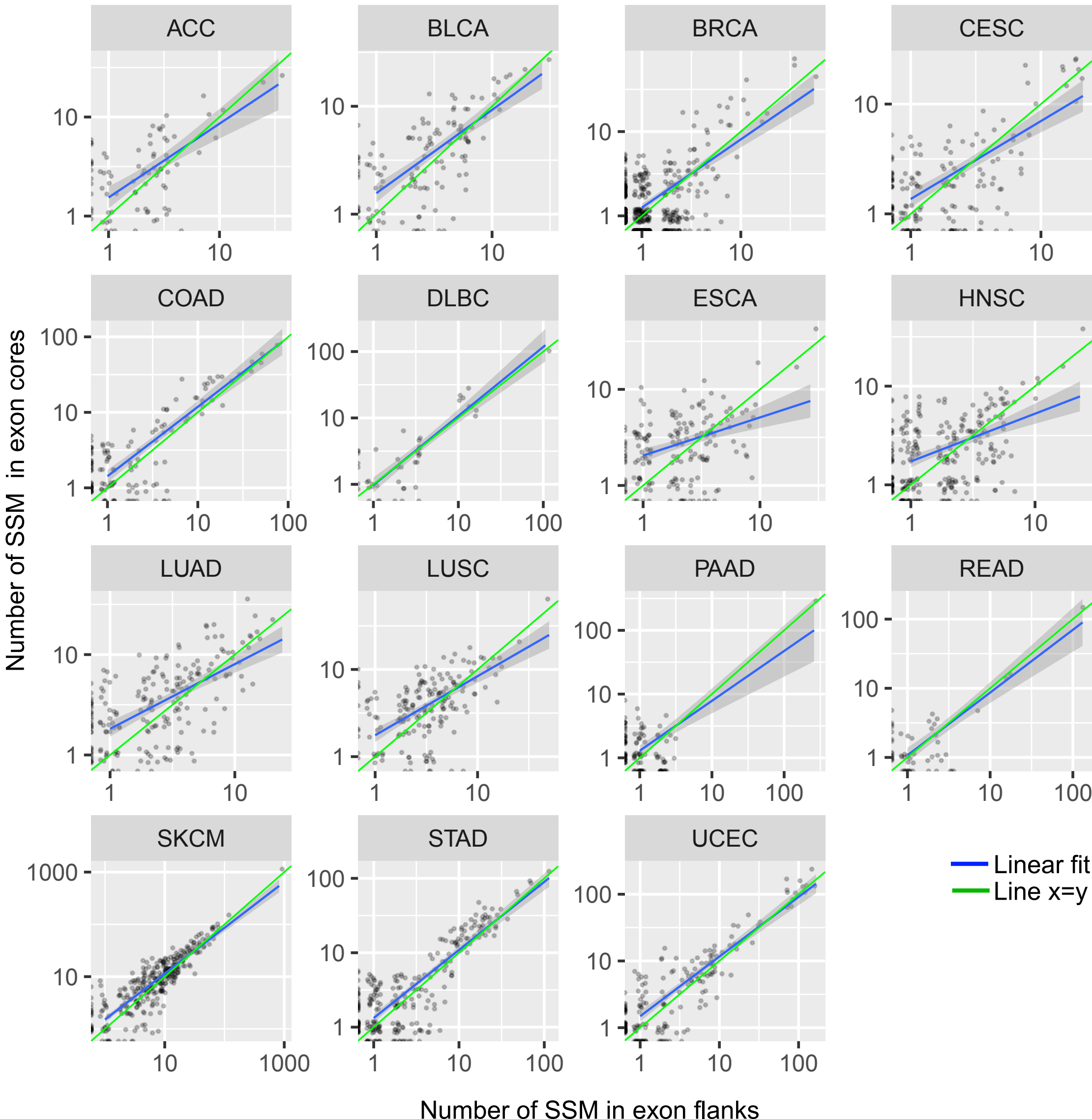

ACC
BLCA
BRCA
CESC
COAD
DLBC
ESCA
HNSC
LUAD
LUSC
PAAD
READ
SKCM
STAD
UCEC
Linear fit
Line x=y
Number of SSM in exon cores
Number of SSM in exon flanks

Figure S4

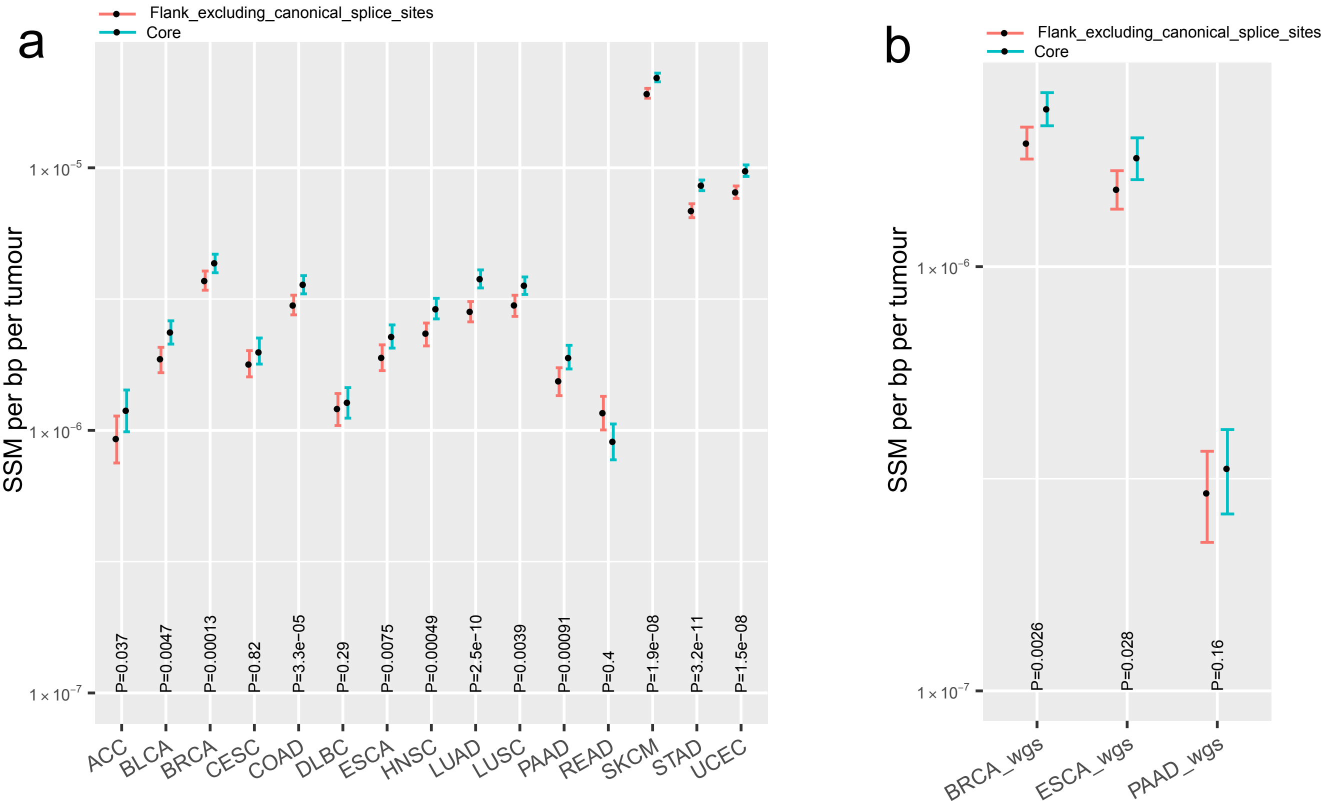

a
Flank_excluding_canonical_splice_sites
Core
SSM per bp per tumour
1 × 10−5
1 × 10−6
1 × 10−7
P=0.037
P=0.0047
P=0.00013
P=0.82
P=3.3e−05
P=0.29
P=0.0075
P=0.00049
P=2.5e−10
P=0.0039
P=0.00091
P=0.4
P=1.9e−08
P=3.2e−11
P=1.5e−08
ACC
BLCA
BRCA
CESC
COAD
DLBC
ESCA
HNSC
LUAD
LUSC
PAAD
READ
SKCM
STAD
UCEC
b
Flank_excluding_canonical_splice_sites
Core
SSM per bp per tumour
1 × 10−6
1 × 10−7
P=0.0026
P=0.028
P=0.16
BRCA_wgs
ESCA_wgs
PAAD_wgs

Figure S5

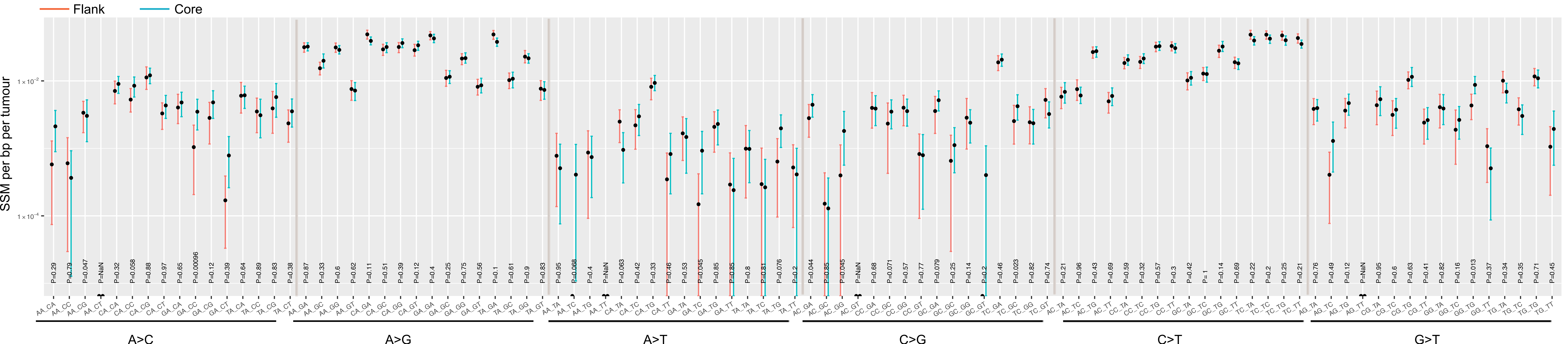

Figure S6

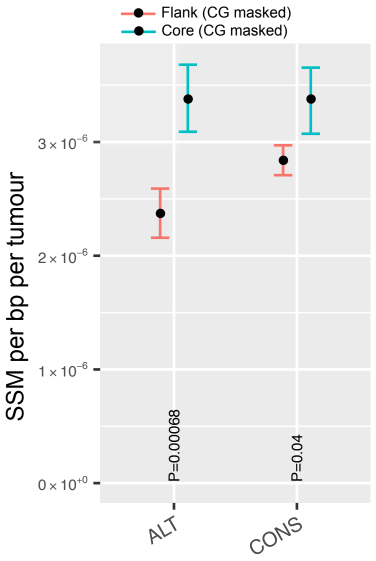

Flank (CG masked)
Core (CG masked)
SSM per bp per tumour
3 × 10⁻⁶
2 × 10⁻⁶
1 × 10⁻⁶
0 × 10⁺⁰
P=0.00068
P=0.04
ALT
CONS

Figure S7

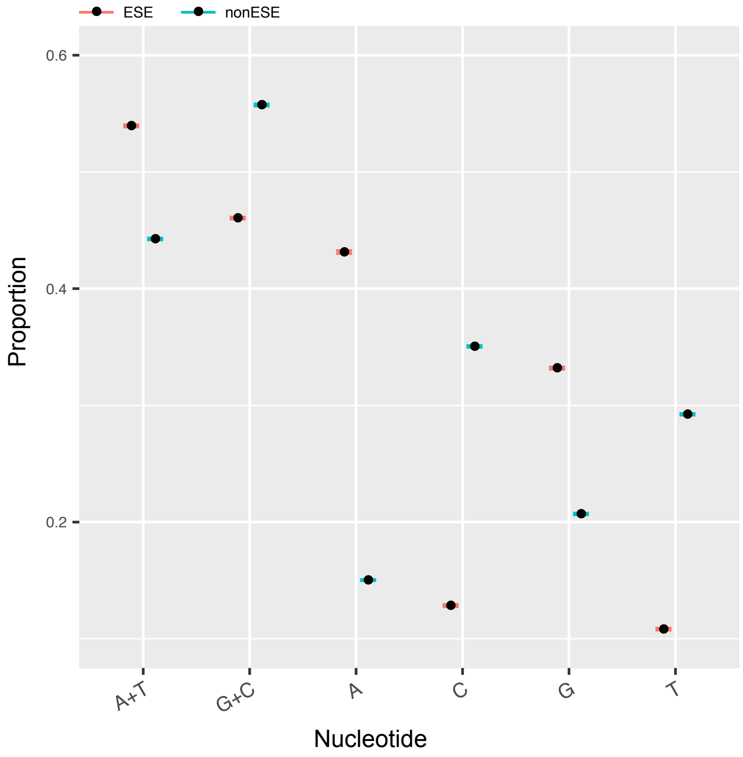

Figure S8

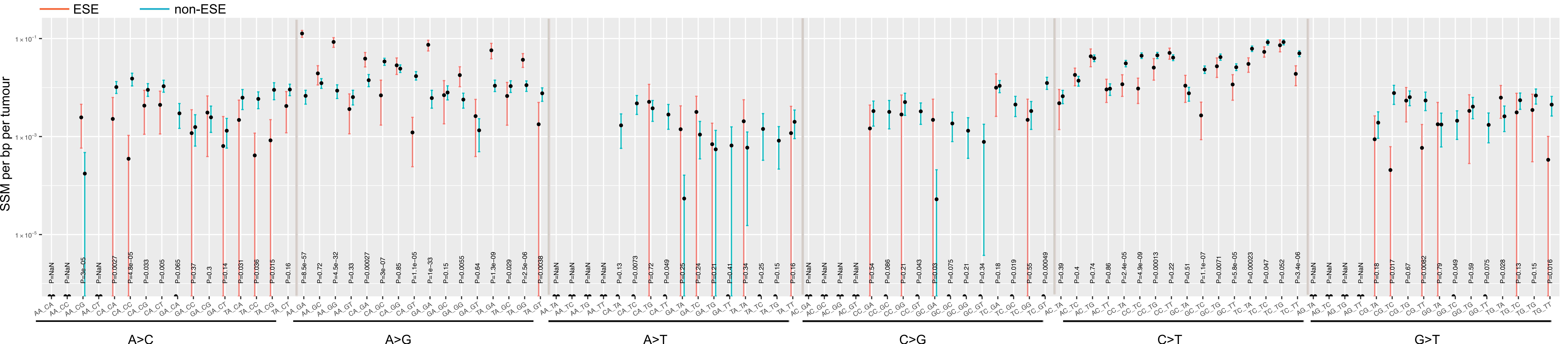

Supplement: Additional file 1: — Supplementary Figures S1–S8. (PDF 1831 kb) [file 13059_2017_1337_MOESM1_ESM.pdf]
